# Research Progress on Extraction, Isolation, Structural Analysis and Biological Activity of Polysaccharides from Panax Genus

**DOI:** 10.3390/molecules28093733

**Published:** 2023-04-26

**Authors:** Shuai Zhang, Chuanbo Ding, Xinglong Liu, Yingchun Zhao, Qiteng Ding, Shuwen Sun, Jinping Zhang, Jiali Yang, Wencong Liu, Wei Li

**Affiliations:** 1 College of Chinese Medicinal Materials, Jilin Agricultural University, Changchun 130118, China; zhangshuai4389@163.com (S.Z.); zhaoyingchun26@163.com (Y.Z.); ssw170331@163.com (S.S.); zjpzjp957@163.com (J.Z.); yjl1104481279@163.com (J.Y.); 2 College of Traditional Chinese Medicine, Jilin Agriculture Science and Technology College, Jilin 132101, China; chuanboding0506@163.com (C.D.); 18844142914@163.com (X.L.); 3 School of Food and Pharmaceutical Engineering, Wuzhou University, Wuzhou 543003, China; 4 College of Life Sciences, Engineering Research Center of the Chinese Ministry of Education for Bioreactor and Pharmaceutical Development, Jilin Agricultural University, Changchun 130118, China

**Keywords:** polysaccharides from Panax genus, extraction, isolation, structural analysis, biological activity

## Abstract

The panax genus is a widely used medicinal plant with good biological activity. As one of the main active components of the Panax genus, polysaccharides have various pharmacological effects. This review summarizes the latest research reports on ginseng, American ginseng, and Panax notoginseng polysaccharides and compares the differences in extraction, isolation and purification, structural characteristics, and biological activities. The current research mainly focuses on ginseng polysaccharides, and the process of extraction, isolation, and structure analysis of each polysaccharide is roughly the same. Modern pharmacological studies have shown that these polysaccharides have antioxidants, antitumor, immunomodulatory, antidiabetic, intestinal protection, skin repair, and other biological activities. This review provides new insights into the differences between the three kinds of ginseng polysaccharides which will help to further study the medicinal value of ginseng in traditional Chinese medicine.

## 1. Introduction

Plants of the genus Panax ginseng are popular for their tonic effects on the human body and serve as an important source of traditional Chinese medicine preparations, health products, functional foods, and cosmetics [1,2,3]. Chinese Pharmacopoeia included four kinds of plants derived from the genus Panax (ginseng; American ginseng; Panax notoginseng; Panax ginseng). At present, there have been more than 6000 reports on ginsenosides [4,5,6] which shows that plants of the genus Panax play an important role in the field of medicine [7]. In recent years, with the development of analytical technology, macromolecular substances have attracted much attention. Polysaccharides have attracted much attention because of their good biological activities in antioxidation, antitumor, and immune regulation [8,9,10]. We searched the PubMed website with the keywords of ginseng polysaccharides and found only reports of ginseng polysaccharides, American ginseng polysaccharides, and Panax notoginseng polysaccharides. Ginseng polysaccharides reported the most, reaching 74.74%, followed by Sanqi polysaccharides at 16.05%; American ginseng polysaccharides at least 9.21% [11,12,13]. First, this paper compared the extraction and separation methods of polysaccharides from plants of the Panax genus. Second, the monosaccharide compositions, molecular weights, and major linkage modes of the three polysaccharides from the Panax genus were characterized by modern techniques. Finally, we reviewed the biological activities of three polysaccharides from the Panax genus. We summarize the research on the polysaccharides of the Panax genus plants to provide a basis for future research on polysaccharides of plants of the Panax genus.

## 2. Separation and Extraction of Polysaccharides from Ginseng

It has been reported that ethanol extraction is used before polysaccharide extraction to remove lipophilic substances and improve the purity of polysaccharides [14]. The extraction methods of polysaccharides in the Panax genus include hot water extraction, alkali extraction, and enzyme extraction [15,16,17]. With the development of ultrasonic and microwave technology, the use of ultrasonic and microwave-assisted extraction not only accelerates extraction efficiency but also greatly improves the extraction rate [18].

At present, hot water extraction and ultrasonic-assisted extraction of polysaccharides from the Panax genus are two commonly used extraction methods. For hot water extraction, Panax genus plants are decocted in boiling water for 2–3 h, and the supernatant is collected by centrifugation and concentrated [19]. The crude extract was precipitated with ethanol at 4 °C for 24 h, and the protein was removed by the Sevage method (a mixture of chloroform and n-butanol at a volume ratio of 4:1) to obtain crude polysaccharides. Ultrasonic-assisted extraction [20] is to break the cell wall under the action of ultrasonic waves, thereby greatly improving the extraction efficiency of polysaccharides. The activity of acidic polysaccharides is easily affected by high temperatures, so NaCO_3_ or KOH is generally used to extract acidic polysaccharides [21]. Enzymes can enzymatically hydrolyze starch granules, thereby increasing the dissolution rate of polysaccharides. Generally, β-amylase and cellulase are commonly used [22].

**Figure 1:**
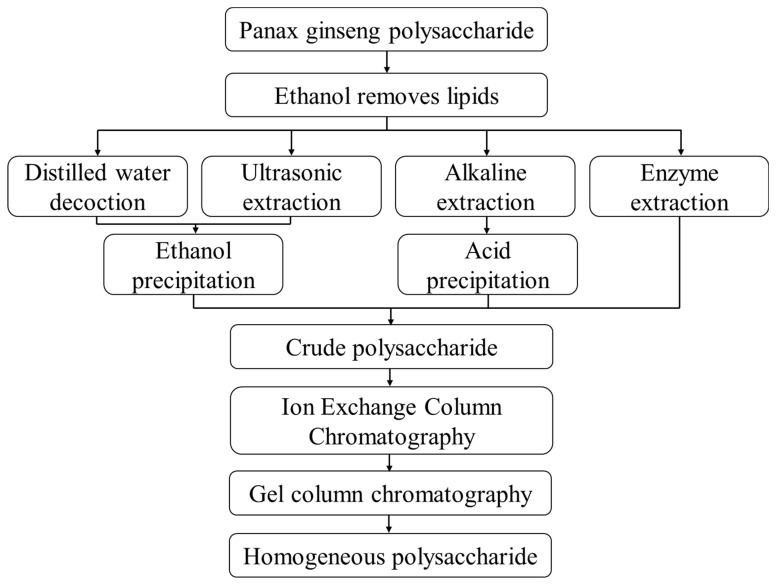
Extraction, separation, and purification process of polysaccharides from Panax genus.

The crude polysaccharide obtained after deproteinization needs to be purified and separated by column chromatography. Common column chromatography methods for separating polysaccharides from the Panax genus include ion-exchange column chromatography and gel column chromatography [23]. Ion-exchange column chromatography is mainly anion-exchange columns of DEAE-cellulose, DEAE-sepharose, and DEAE agarose gel which are used for the separation of neutral polysaccharides and acidic polysaccharides [24]. Gel column chromatography is mainly dextran and agarose gel columns, and polysaccharides are separated according to their molecular weight and shape [25]. The commonly used method is the combined application of an ion exchange column and gel chromatography column to separate and purify the polysaccharides of the Panax genus [26]. After dialysis (beneficial to desalination, dealcoholization, and removal of small molecule polysaccharides and other aqueous impurities) and then freeze drying, a homogeneous polysaccharide component is obtained. The flow chart of the extraction and separation of polysaccharides from the Panax genus is shown in Figure 1.

## 3. Structural Analysis of Polysaccharides from the Panax Genus

Panax ginseng polysaccharides are natural polymers composed of various monosaccharides linked to each other by glycosidic bonds. To analyze its structural characteristics, physical and chemical methods are generally used to characterize the primary structure of polysaccharides, including molecular weight, monosaccharide composition, and glycosidic linkages. 

### 3.1. Ginseng

Among the plants of the Panax genus, the research on ginseng polysaccharides is the main one, and its structure is also the most abundant (Table 1). Li [27] extracted water-soluble ginseng polysaccharides (WGP) from ginseng roots and then purified them by ion-exchange chromatography to obtain neutral components (WGPN) and acidic components (WGPA). The monosaccharide composition was analyzed by HPLC, and it was found that there was a large difference in the monosaccharide composition of the two components. A study proposed a new two-dimensional correlation infrared spectroscopy (2DCOS-IR) method for the identification of ginseng polysaccharides [28]. 2D-sATR-FTIR has the advantages of high throughput and high efficiency in polysaccharide quality evaluation of ginseng polysaccharides. It not only enriches the identification method of ginseng polysaccharides but also establishes the prediction model of ginseng polysaccharides by using stoichiometric analysis. The structural characteristics of MCGP-L were studied by a combination of chemical and instrumental analysis [29]. MCGP-L is composed of glucose, galactose, and mannose in a ratio of 14.8:1:1.2, and its main chain is composed of (1→4)-α-D-Glcp with branches at (1→4, 6)-α-O-6 position substitution.

Ginseng is mainly composed of neutral sugars, but there were also a small part of acidic sugars. Kim [30] extracted and separated neutral and acidic polysaccharides from ginseng, and their monosaccharide composition contained less galactose and glucose and higher galacturonic acid and glucuronic acid. Jia [31] isolated (MCG) polysaccharides from wild ginseng and obtained seven acidic polysaccharides (MCGP-1-MCGP-7) through further separation. The structure was characterized by HPLC, HPGPC, GC-MS, and NMR; the molecular weight and monosaccharide composition of seven polysaccharides were compared, and the main chain and branch chain structures of MCGP-3 and MCGP-4 were identified.

Ginseng polysaccharides exist not only in the rhizomes of ginseng but also in the stems, leaves, and berries of ginseng [32,33]. Ginseng polysaccharides composed of glucose, galactose, arabinose, and rhamnose were extracted from ginseng berries [34]. When ginseng is processed into red ginseng, there will be a Maillard reaction, and the high temperature will break the sugar chain, so the structure of red ginseng polysaccharides is different from that of ginseng polysaccharides. After acid and alkali treatment, the monosaccharide composition will also change and affect the polysaccharide structure [35]. Jin [36] established a matrix-assisted laser desorption/ionization time-of-flight/time-of-flight mass spectrometry method for analyzing the structure of red ginseng polysaccharides which provides a new method for the study of polysaccharide structures.

### 3.2. American Ginseng

The research on American ginseng by Chinese people is relatively late, especially the research on the structure of polysaccharides is less (Table 2). Some studies extracted American ginseng crude polysaccharide (AGPS) with hot water and used resin S-8 and polyamide column for preliminary purification, and then used DEAE-Sepharose CL-6B and Sepharose CL-6B column chromatography for further purification and separation to obtain five species polysaccharide components. They compared the structural features of the above polysaccharides using UV-Vis spectroscopy, HPGFC, GC, SEM, IR, and NMR methods [12]. Wang [37] isolated a new polysaccharide with a molecular weight of 3.1 kDa from American ginseng which was composed of glucose (Glc) and galactose (Gal) with a molar ratio of 1:1.15. Yu [38] used 0.3 mol/L NaOH to extract two polysaccharides from American ginseng roots. AEP-1 was composed of Glc, Gal, GalA, and AEP-2 mainly contained Ara, Man, Gal, Glc, and GalA. Some studies have used ultrasound-assisted extraction of American ginseng polysaccharides which are composed of Ara, Rha, GalA, Man, Glc, and Gal in a ratio of 31:4:1:2:72:59, mainly composed of→4)-GalA-(1→[39]. According to the current research, the research on polysaccharides of American ginseng needs to be deepened, and the next step should be to clarify the chemical structure of polysaccharides of American ginseng based on analytical techniques.

### 3.3. Panax notoginseng

As a traditional Chinese medicine, Panax notoginseng plays an important role in promoting blood circulation and removing blood stasis. The focus of Panax notoginseng is more on ginsenosides and Panax notoginseng polysaccharide, as a non-saponin component, also plays an important role (Table 3). Some studies extracted water-soluble polysaccharides from Panax notoginseng obtained an amyloid polysaccharide and six pectin components [13] and characterized their structures by combining monosaccharide composition, enzymatic hydrolysis, NMR, and methylation analysis. The six pectins belonged to the types AG-II, RG-I, HG, and RG-II, and the structure of Panax notoginseng polysaccharides was analyzed. Wu [40] isolated a kind of arabinoglucogalactan from Panax notoginseng, oxidized it with NaIO4 and CrO3, degraded it with Smith, hydrolyzed it with graded acid, identified its structure by spectrum, and obtained it as (1→3)-β-D-Gal is the main chain polysaccharide. Liu [41] extracted the residue of Panax notoginseng to realize the full development of Panax notoginseng resources, separated and obtained six kinds of Panax notoginseng polysaccharides, and carried out a structural analysis on each component, acidic *Panax notoginseng* (APPN)II-B and APPN III-B belong to HG type pectin. Wang [42] extracted a structure from Panax notoginseng which was determined to have a main chain of 1,6-linked Galp, branched by 1,3-linked Galp at C3, and the branch was connected at its O-3 Position of the arabinogalactan RN1.

## 4. Biological Activity of Ginseng Polysaccharides

Panax ginseng polysaccharides, as one of the main active substances in ginseng plants, have attracted much attention for their good biological activities and play important roles in antioxidation, antitumor, and immune regulation. The biological activities of the polysaccharides of the Panax genus are now shown in Table 4.

### 4.1. Antioxidant Effect

Panax ginseng polysaccharides play an important role in antioxidants due to their unique structure. Zhao [43] isolated MCGOS-70 and MCGOS-95 from ginseng, and both showed good antioxidant activity by measuring the ABTS free radical scavenging rate, DPPH free radical scavenging rate, and ferric iron-reducing ability. The structure of polysaccharides in different parts of the same plant is different, and their antioxidant capacity is also affected. By comparing DPPH free radical scavenging rate, hydroxyl free radical activity, and ferrous ion chelating ability, it was found that the in vitro antioxidant activity of ginseng root polysaccharides was higher than that of ginseng flower and leaf polysaccharides [44]. Ginseng roots polysaccharides increase superoxide dismutase (SOD), catalase (CAT), glutathione peroxidase (GSH-Px) and total antioxidant capacity (T-AOC) in mouse serum and liver) activity and reduce the level of malondialdehyde (MDA) to play an antioxidant role. Chen [11] extracted polysaccharides from ginseng rhizomes, evaluated the antioxidant activity by measuring the scavenging rate of DPPH free radicals, and found that the antioxidant activity of the aerial part was stronger than that of the underground part. The antioxidant activity of aerial neutral polysaccharides was stronger than that of acidic polysaccharides. By measuring the ABTS free radical scavenging rate and oxygen free radical absorption capacity of American ginseng polysaccharides, it was shown that it has good antioxidant activity [38]. Panax notoginseng polysaccharide also has a high DPPH free radical scavenging ability [40]. It can be seen that ginseng polysaccharides have a strong antioxidant capacity and are expected to become natural antioxidants.

### 4.2. Antitumor Effect

Ginseng berry polysaccharides can promote mouse peritoneal macrophage activation and NK cell cytotoxicity and dose-dependently increase anticomplement activity and cytokine production, including interleukin (IL)-6, IL-12, and tumor necrosis factor (TNF)-α. By taking ginseng berry polysaccharide orally, the cancer inhibition rate was 37% while by intravenous injection of ginseng berry polysaccharide, the cancer inhibition rate could reach 48% [45]. Ginseng leaf polysaccharide exhibited antitumor activity by promoting the activation of macrophages and NK cells [46] and was also able to promote the secretion of TNF-α and IL-12 in mouse macrophages. In addition, mouse splenocytes treated with ginseng leaf polysaccharide significantly enhanced the cytotoxicity of NK cells against YAC-1 tumor cells. American ginseng polysaccharides are cytotoxic to HT29 cancer cells and can cause HT29 cancer cells to have a significant decrease in cell number, cell cycle arrest in G2/M, increased cell death, and increased expression of cleaved caspase-3. Panax notoginseng crude polysaccharide can effectively prolong the lifespan of tumor-bearing mice by enhancing the host immune system and weak cytotoxicity to liver cancer cells [47]. The neutral polysaccharide isolated from Panax notoginseng crude polysaccharide can not only inhibit the growth of H22 cells but also significantly increase the tumor inhibition rate of tumor-bearing mice in combination with cyclophosphamide (CTX) [48].

### 4.3. Immunomodulatory Effect

Macrophages play an important role in adaptive and innate immunity, thereby regulating the immune system. Red ginseng polysaccharides had no cytotoxicity to RAW264.7 macrophages and promoted the phagocytosis of macrophages and the release of NO [49]. Ginseng flower polysaccharides can enhance the phagocytosis of RAW264.7 macrophages and can promote the release of NO and increase the secretion of TNF-α, IL-6, gamma interferon (IFN-γ), and IL-1β. It can also enhance in vivo immunity in CTX-induced immunosuppressed mice [50]. Alveolar macrophages extracted from rats treated with American ginseng polysaccharides increased NO production. By detecting the TNF-α content in rat plasma, it was found that the TNF-α content of the American ginseng polysaccharide group was higher than that of the blank group [51]. Panax notoginseng polysaccharide can enhance complement fixation activity and activate polymorphonuclear neutrophils to generate reactive oxygen species (ROS) for mitogenic effect [52]. Studies have also shown that Panax notoginseng polysaccharide can promote the release of IFN-γ and TNF-α from mouse spleen lymphocytes and peritoneal macrophages for immune regulation. Panax ginseng plant polysaccharides mainly enhance the proliferation of immune cells (lymphocytes, macrophages, NK cells, and dendritic cells) and promote the release of various cytokines (IL-6, IL-12, IFN-γ, and TNF-α) which play a role to immunomodulation.

### 4.4. Antidiabetic Effect

Diabetes is a global social disease affecting millions of people. As a natural medicine, ginseng polysaccharide can reduce the level of MDA in the serum of streptozotocin-induced mice and increase the serum insulin, SOD activity, and liver glycogen level [53]. At the same time, ginseng polysaccharides can reverse the dysregulated intestinal flora of diabetic rats, upregulate the relative abundance of Bacteroides, increase fecal β-D-glucosidase activity, and enhance the hypoglycemic effect of ginsenosides [54]. American ginseng fructose polysaccharide has strong activity in reducing fasting blood glucose and improving glucose tolerance in mice [55]. Suzuki Y [56] studied the mechanism of ginseng polysaccharides Panaxan A and Panaxan B in lowering blood sugar. The results showed that Panaxan A did not affect the activity of liver glucokinase but could increase the activity of glucose-6-phosphatase; Panaxan B did not affect the activity of these enzymes but decreased the activity of liver glycogenase.

### 4.5. Intestinal Protection

Inflammatory bowel disease, which includes Crohn’s disease and ulcerative colitis, is a relapsing, refractory gastrointestinal disorder [57]. Ginseng polysaccharides restore mTOR-dependent autophagy dysfunction by modulating gut microbiota structure and blocking the TLR4–MyD88 pathway. Autophagy inhibits inflammation by inhibiting NF-κB, oxidative stress and cytokine release [58]. Ginseng polysaccharides can promote the recovery of the intestinal mucosal structure by regulating intestinal flora, increasing the number of beneficial bacteria, and balancing metabolic processes [59]. Ginseng acidic polysaccharides protect the mouse’s small intestine from radiation-induced damage by prolonging crypt cell proliferation and reproduction of villi [60]. Investigating its molecular mechanism found that ginseng acidic polysaccharides protected mouse small intestine from radiation-induced apoptosis by inhibiting p53-dependent pathways and mitochondrial/caspase pathways [61]. American ginseng polysaccharide combined with ginsenoside can upregulate the ratio of villi height/crypt depth, mucin expression area, goblet cell number, and tight junction protein expression. By modulating gut microbiota composition and various metabolites, CTX-induced intestinal immune disturbance and intestinal barrier dysfunction were improved [62].

### 4.6. Skin Repair

**Figure 2:**
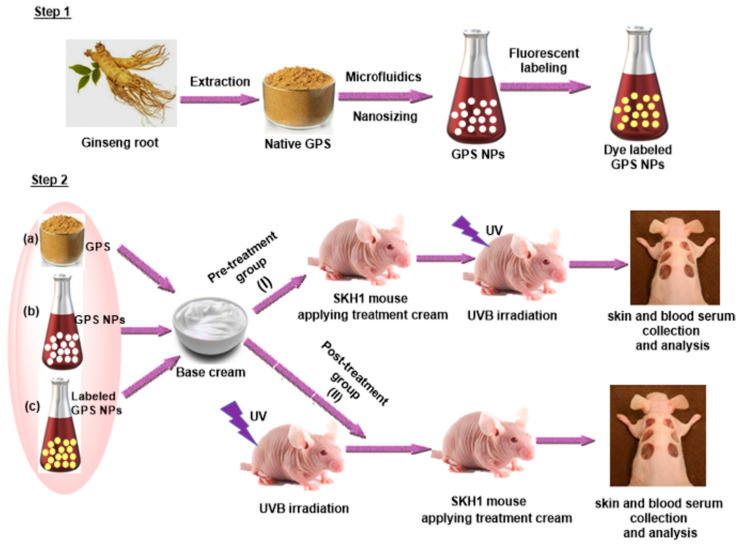
Schematic illustration of experimental design and analysis. (step 1): extraction, nanosizing and labeling of GPS and (step 2): photo-protective effect of GPS on SKH1 hairless mice through two different routes (I) pre-treatment with GPS and (II) post-treatment with GPS. Three different treatment groups were used (a) native GPS, (b) GPS NPs, and (c) labeled GPS NPs.

Skin injury repair is a complex dynamic regulation process involving a series of temporally and spatially ordered interactions of cells, cytokines, and extracellular matrix. We can artificially divide the repair process of skin damage into four overlapping phases, namely blood coagulation phase, inflammation phase, proliferation phase, and tissue remodeling phase. Dysregulation of one or more stages in the injury healing process will result in chronic non-healing wounds or pathological scarring. Ginseng oligosaccharides repair the skin barrier damage caused by UVB by alleviating the symptoms of skin dryness and desquamation, exerting its potential as a natural cosmetic [63]. Acidic polysaccharides, as functional polysaccharides in red ginseng, repair skin damage by stimulating activator protein-1 and inhibiting solar ultraviolet-induced matrix metalloproteinase-1 protein expression [64]. In particular, pre-treatment with GPS gave better protection against visible changes (wrinkling), histological alterations and cytokine production compared to posttreatment with GPS. Topical formulations of American ginseng polysaccharide nanoparticles can reduce the levels of pro-inflammatory cytokines and reduce UVB-induced oxidative damage and skin cancer by inhibiting the initiation of pro-inflammatory cascades [65] (Figure 2).

### 4.7. Other Activities

In addition to the above biological activities, other biological functions of Panax ginseng polysaccharides have also been reported. Steamed ginseng polysaccharide can prolong the exhausted swimming time of fatigued mice, increase liver and muscle glycogen levels, superoxide dismutase, catalase, glutathione peroxidase activities, and reduce serum lactic acid, nitrogen and MDA levels [66]. Ginseng polysaccharides reduce renal cell apoptosis by inhibiting the PERK-eIF2α-ATF4 signaling pathways activated by endoplasmic reticulum stress caused by cisplatin, thereby improving nephrotoxicity [67]. Liu [19] found that ginseng polysaccharides prevented ethanol-induced gastric injury in rats by inhibiting gastric inflammation and oxidative stress through NF-κB and STAT. Ginseng polysaccharides can exert anti-immune aging effects by inhibiting thymus degeneration and regulating various types of immune cells [68]. Panax notoginseng acidic polysaccharides can improve alcohol-induced liver injury in mice by enhancing the alcohol dehydrogenase (ADH) pathway and inhibiting the catalase pathway of ethanol metabolism to prevent the accumulation of peroxides [69].

## 5. Conclusions

This article summarizes the recent progress on polysaccharides from the Panax genus in recent years and compares the differences in extraction, isolation, and structural characteristics. Most studies have focused on ginseng polysaccharides with less research on American ginseng and Panax notoginseng. Panax ginseng polysaccharides have antioxidant activity, antitumor activity, immunomodulatory effect, antidiabetic activity, intestinal protective activity, skin repair, and other pharmacological functions mediated by various signals, including MAPK, NF-κB, and redox pathways.

The study of plants in the genus Panax remains a hot topic due to their massive consumption worldwide in many fields, such as medicine, functional food, nutraceuticals, and cosmetics. Panax genus polysaccharides, as one of the active components of Panax genus plants, have benefited from the continuous development of analytical techniques, such as HPLC, GC-MS and NMR, to fully explain the chemical basis among different Panax genus species. As the structure of polysaccharides from the Panax genus has gradually been uncovered, more attention has been paid to its good biological activity. Panax ginseng polysaccharides, which are natural plant sources, will be used more widely.

Panax ginseng polysaccharides, as non-medicinal parts of ginseng plants, have very good biological activity. Through the analysis and summary of this article, we can understand their structure and biological activity more intuitively and provide a basis for their future utilization and development. At the same time, the development and utilization of polysaccharides from plants of the genus Panax can drive the comprehensive development and application of plants of the genus Panax and provide a basis for future development.

## Figures and Tables

**Table 1:** Structural features of ginseng polysaccharides.

Serial Number	Polysaccharide Name	Monosaccharide Composition and Ratio	Molecular Weight	Structure
1	WGPN	Glc:Gal:Ara =95.3:3.3:1.4		
2	WGPA	Glc:Gal:Ara:GalA:Rha = 13.6:18:15.4:44.2:3.8		Contains RG-I and HG
3	MCGP-3	Glc:Gal:GlcA:GalA:Rha:Man:Ara = 33.17:22.88:0.687:15.67:6.005:0.631:20.96	1.572 × 10^5^	RG-I
4	MCGP-4	Glc:Gal:GlcA:GalA:Rha:Man:Ara = 7.146:39.74:1.519:26.74:4.533:0.214:20.11	1.673 × 10^5^	RG-I
5	MCGPL	Glc:Gal:Man = 14.8:1:1.2	3 × 10^3^	The main chain is composed of (1→4)-α-D-Glcp
6	WGNP	Glc:Gal:Ara = 97.9:1.1:1	16.1–70.4 × 10^3^	
7	WGAP	Glc:Gal:GlcA:GalA:Ara = 24:24.4:32.2:1.3:18.1	50–80 × 10^3^	
8	GFP1	Glc:Gal:Ara:Rha = 2:6.1:3.2:1.1	1.4 × 10^5^	The main chain is composed of (1→6)-Galp and (1→3,6)-.
9	RGCW-EZ-CP-4	Gal:Ara:GalA = 29.9:19.8:38.6		Contains RG-I and RG-II
10	RG-CW-EZ-CP-8	Gal:GalA:Ara = 10.3:12.3:64.3		The main chain is arabinan or arabinogalactan.

**Table 2:** Structural characteristics of American ginseng polysaccharides.

Serial Number	Polysaccharide Name	Monosaccharide Composition and Ratio	Molecular Weight	Structure
1	WPS-1	Ara:Rha:Man:Gal:Glc = 21.2:2.3:2.6:18.7:5.2	1.54 × 10^6^	Mainly composed of (1→6)-α-D-Glcp and (1→5)-α-L-Araf
	WPS-2	Ara:Rha:Man:Gal:Glc = 7.9:1.7:2.9:20.7:46.8	1.41 × 10^4^	
3	SPS-1	Ara:Xyl:Man:Gal:Glc:GalA:GlcA = 2.3:6.9:9.2:28.6:15.9:13.6:3.5	3.62 × 10^5^	Mainly composed of (1→6)-α-D-Glcp, (1→4)-α-D-Manp, (1→5)-α-L-Araf, β-D-Galp and β-D-xylose RG-I.
4	SPS-2	Ara:Xyl:Man:Gal:Glc:GalA:GlcA = 14.2:5.3:7.9:22.5:25.3:16.9:7.9	9.7 × 10^5^	
5	SPS-3	Ara:Rha:Xyl:Man:Gal:Glc:GalA:GlcA = 19.2:2.1:9.6:12.0:15.2:11.5:26.3:4.1	5.12 × 10^5^	
6	PPQN	Glc:Gal = 1:1.15	3.1 × 10^3^	
7	AEP-1	Glc:Gal:GalA = 4.67:0.97:3.92		
8	AEP-2	Ara:Man:Gal:Glc:GalA = 1.03:0.76:1.68:3.02:3.65		

**Table 3:** Structural characteristics of Panax notoginseng polysaccharide.

Serial Number	Polysaccharide Name	Monosaccharide Composition and Ratio	Molecular Weight	Structure
1	PNPA-1A	GalA:Rha:Gal:Ara:Glc:Man = 5:0.8:63.2:27.7:2.4:0.9	8.8 × 10^4^	AG-Ⅱ Mainly HG, composed of different proportions of RG-I and RG-II.
2	PNPA-1B	GalA:Rha:Gal:Ara:GlcA:Man = 11.6:6:46:33.4:1:2	1.01 × 10^5^	
3	PNPA-2A	GalA:Rha:Gal:Ara:Glc:Man = 15.9:15.5:32.7:28.3:2.2:4	2.7 × 10^5^	AG-Ⅱ Mainly HG, composed of different proportions of RG-I and RG-II. RG-Ⅰ
	PNPA-2B	GalA:Rha:Gal:Ara:Glc:GlcA:Man = 40.6:9.6:29.3:10.4:4.5:0.6:2.9	3 × 10^3^	
5	PNPA-3A	GalA:Rha:Gal:Ara:GlcA:Man = 74.4:7.5:8.3:8.2:0.8:0.8	6 × 10^3^	
6	PNPA-3B	GalA:Rha:Gal:Ara:Glc:GlcA:Man = 75.8:5.2:8.8:5.1:1.6:0.9:1.4	1.8 × 10^4^	Mainly HG, composed of different proportions of RG-I and RG-II
7	Arabinogalactan	Ara:Glc:Gal = 1:1:8	6.7 × 10^4^	(1→3)-β-D-galactosyl residue is the backbone, α-L-Araf-(1→4)-β-D-Glcp-(1→is the branch.
8	NPPN	Ara:Gal:Glc:Man = 3.76:18.58:76.85:0.80	2.3 × 10^5^	
9	APPN-Ⅰ	Ara:Gal:Glc:Man:GalA:GlcA = 11.47:34.82:43.48:2.28:5.66:2.29	4.9 × 10^5^	The main chain is composed of α-1,4-Glcp glycosidic linkages.
10	APPNⅡ-A	Ara:Gal:Glc:GalA:GlcA = 11.04:39.59:39.80:7.03:2.54	4.5 × 10^5^	The main chain is composed of α-1,4-Glcp glycosidic linkages.
11	APPNⅡ-B	Ara:Gal:Glc:GalA = 1.49:1.64:2.50:94.36	2.8 × 10^4^	HG
12	APPNⅢ-A	Fuc:Ara:Gal:Glc:Xyl:Man:GalA:GlcA = 1.61:9.45:39.25:16.61:1.11:1.74:26.66:3.57	3.4 × 10^5^	Linked by β-pyranoside.
13	APPNⅢ-B	Ara:Gal:Glc:GalA = 1.22:1.52:2.90:94.36	5.6 × 10^4^	HG
14	RN1	Gal:Ara = 43.7:56.3	2.1 × 10^4^	Consists of 1,6 linked Galp residues.

**Table 4:** Biological activities of panax ginseng polysaccharides.

Serial Number	Source Plant	Biological Activity	Animal Model	Molecular Mechanism
1	ginseng	Anti-oxidation	In vitro	Determination of ABTS free radical scavenging rate, DPPH free radical scavenging rate, and ferric iron reducing ability
2	ginseng	Anti-oxidation	D-Gal-induced ICR mice	Increases the activity of SOD, CAT, GSH-Px, and T-AOC in mouse serum and liver, and reduces the level of MDA to play an antioxidant role
3	ginseng	Anti-oxidation	In vitro	Determination of DPPH free radical scavenging rate
4	American ginseng	Anti-oxidation	In vitro	Determination of ABTS free radical scavenging rate and oxygen free radical absorption capacity
5	Panax notoginseng	Anti-oxidation	In vitro	Determination of DPPH free radical scavenging rate
6	ginseng	Antitumor	B16-BL6 melanoma cells implanted in female BALB/c mice	Increased release of IL-6, IL-12, TNF-α, IFN-γ, and granzyme B from NK cells to inhibit tumor aggregation
7	ginseng	Antitumor	Colon 26-M3 cells and BALB/c mice	Promote the activation of macrophages and NK cells to play an antitumor role
8	American ginseng	Antitumor	HT29 cells	Inhibits cancer cell growth by causing decreased cell number, cell cycle arrest at G2/M, increased cell death, and increased expression of cleaved caspase-3
9	Panax notoginseng	Antitumor	HT22 cells and tumor-bearing mice	Antitumor effect by enhancing host immune system and weak cytotoxicity against liver cancer cells
10	Panax notoginseng	Antitumor	HT22 cells and tumor-bearing mice	Inhibit the growth of H22 cells, combined with CTX to increase the tumor inhibition rate of tumor-bearing mice
11	ginseng	Immunomodulatory	RAW264.7 macrophages	Promote the phagocytosis of macrophages and the release of NO
12	ginseng	Immunomodulatory	RAW264.7 macrophages	Increased TNF-α, IL-6, IFN-γ, and IL-1β levels and release of NO
13	ginseng	Immunomodulatory	CTX-induced BALB/c mice	Enhance immunity by activating macrophages
14	American ginseng	Immunomodulatory	LPS-induced rats	Increased TNF-α level and NO release from isolated alveolar macrophages
15	Panax notoginseng	Immunomodulatory	Human polymorphonuclear neutrophils	Enhancing complement fixation activity and promoting mitosis by regulating ROS and IFN-γ
16	Panax notoginseng	Immunomodulatory	Mouse spleen lymphocytes and peritoneal macrophages	Induces production of interferon-γ and TNF-α
17	ginseng	Antidiabetic	STZ-induced ICR mice	Reduce serum MDA level, increase serum insulin, SOD activity, and liver glycogen level
18	ginseng	Antidiabetic	STZ-induced rats	Upregulates the relative abundance of Bacteroides and increases fecal β-D-glucosidase activity
19	American ginseng	Antidiabetic	ob/ob mice	Reduce fasting blood glucose in mice
20	ginseng	Gut protection	SD rats induced by DSS	Regulation of intestinal flora structure and blocking of TLR4-MyD88 pathway to inhibit NF-κB, oxidative stress, and cytokine release inhibit inflammation
21	ginseng	Gut protection	Balb/c mice induced by lincomycin hydrochloride	Regulate the number of intestinal flora, balance the metabolic process
22	ginseng	Gut protection	Irradiated C57BL/6 mice	Inhibition of p53-dependent and mitochondrial/caspase pathways reduces apoptosis.
23	American ginseng	Gut protection	CTX-induced C57BL/6 mice	Regulating gut microbiota and metabolites
24	ginseng	Skin repair	NC/Nga mice	Suppression of solar ultraviolet-induced matrix MMP-1 protein expression by stimulating AP-1
25	American ginseng	Skin repair	SKH1 hairless mice.	Reduces the level of pro-inflammatory cytokines and inhibits the initiation of pro-inflammatory cascades

## Data Availability

Publicly available datasets were analyzed in this study. This data can be found here: https://pubmed.ncbi.nlm.nih.gov/.

## Abbreviations

SOD: superoxide dismutase; CAT, catalase; GSH-Px, glutathione peroxidase; T-AOC, total antioxidant capacity; MDA, malondialdehyde; IL-6, interleukin; TNF-α, tumor necrosis factor-α; IFN-γ, gamma interferon; ROS, reactive oxygen species; CTX, cyclophosphamide; ADH, alcohol dehydrogenase.
